# Supplementary material for: Naturally-derived protein extract from Gryllus bimaculatus improves antioxidant properties and promotes osteogenic differentiation of hBMSCs
Source: PLoS One. 2021 Jun 2;16(6):e0249291. doi: 10.1371/journal.pone.0249291 (PMC8172014; doi:10.1371/journal.pone.0249291)
Supplement: S1 Fig — (DOCX) [file pone.0249291.s001.docx]

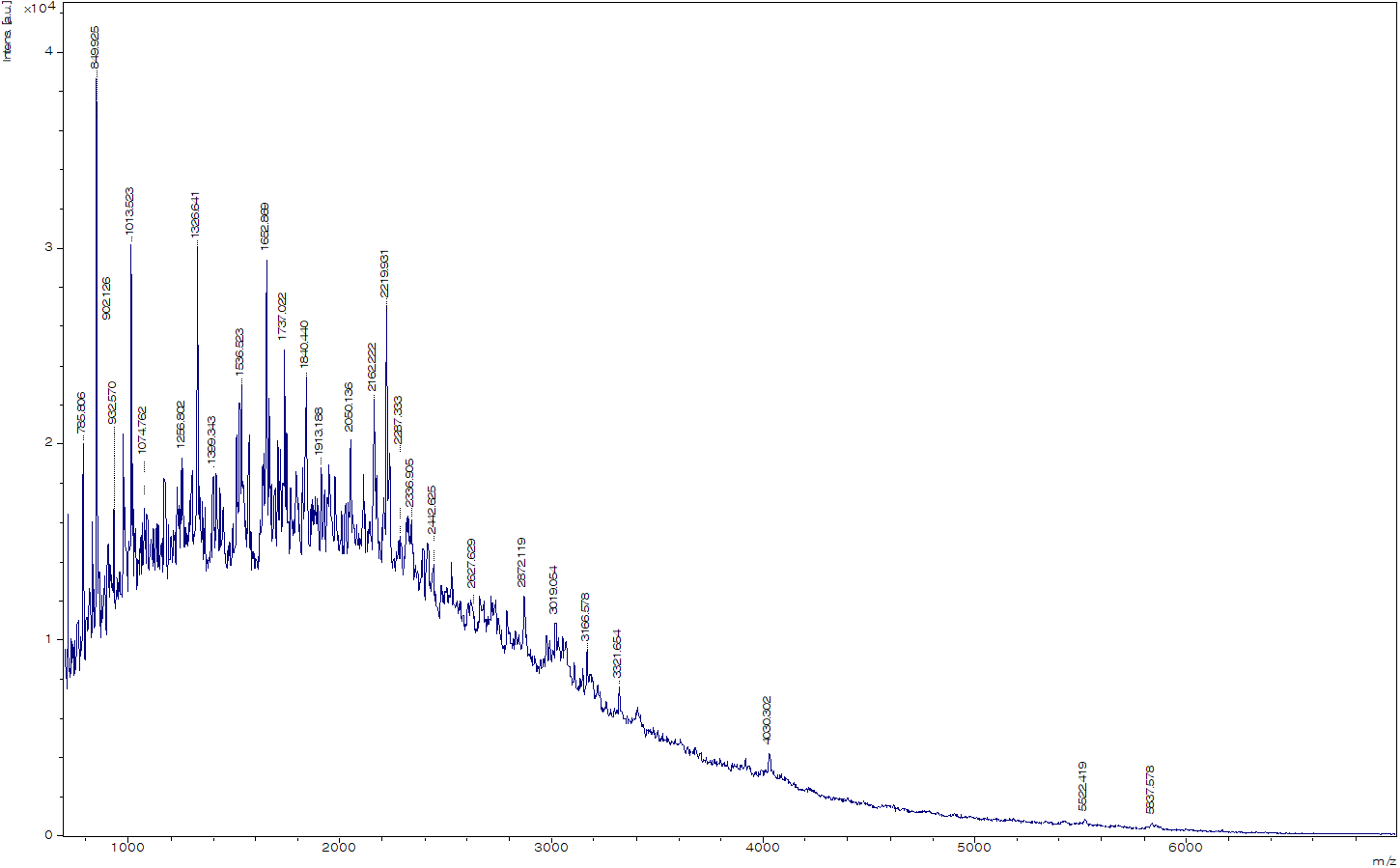


**S1 Fig.** MALDI TOF MS spectra of CPI for the determination of molecular weight of peptides below 6kDa*.*
