# Supplementary material for: Naturally-derived protein extract from Gryllus bimaculatus improves antioxidant properties and promotes osteogenic differentiation of hBMSCs
Source: PLoS One. 2021 Jun 2;16(6):e0249291. doi: 10.1371/journal.pone.0249291 (PMC8172014; doi:10.1371/journal.pone.0249291)
Supplement: S2 Fig — (DOCX) [file pone.0249291.s002.docx]

**S2 Fig.** Cell death percentage in control and 2% CPI.
