# Supplementary material for: Naturally-derived protein extract from Gryllus bimaculatus improves antioxidant properties and promotes osteogenic differentiation of hBMSCs
Source: PLoS One. 2021 Jun 2;16(6):e0249291. doi: 10.1371/journal.pone.0249291 (PMC8172014; doi:10.1371/journal.pone.0249291)
Supplement: S3 Fig — Arrowhead indicates the presence of CPI aggregates deposited on the surface of cells. (DOCX) [file pone.0249291.s003.docx]

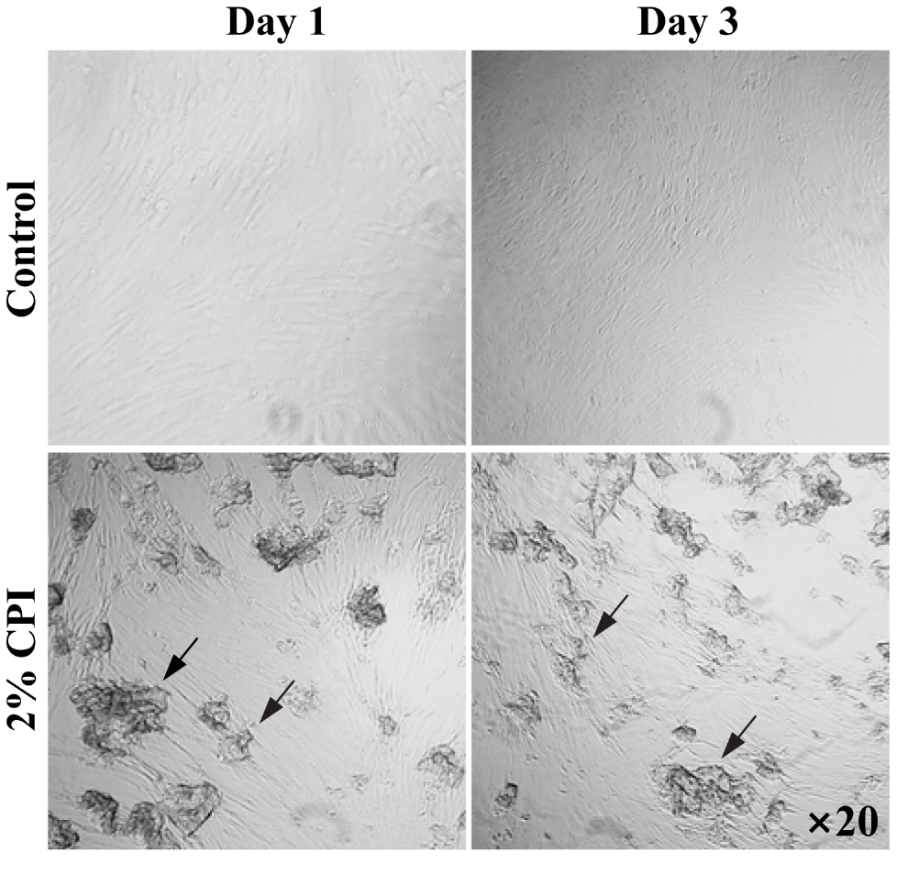


**S3 Fig.** Representative bright-field images of CPI-treated hBMSCs at indicated time intervals (Magnification ×20). Arrowhead indicates the presence of CPI aggregates deposited on the surface of cells.
