# Supplementary figures and images for: Naturally-derived protein extract from Gryllus bimaculatus improves antioxidant properties and promotes osteogenic differentiation of hBMSCs
Source: PLoS One. 2021 Jun 2;16(6):e0249291. doi: 10.1371/journal.pone.0249291 (PMC8172014; doi:10.1371/journal.pone.0249291)

**
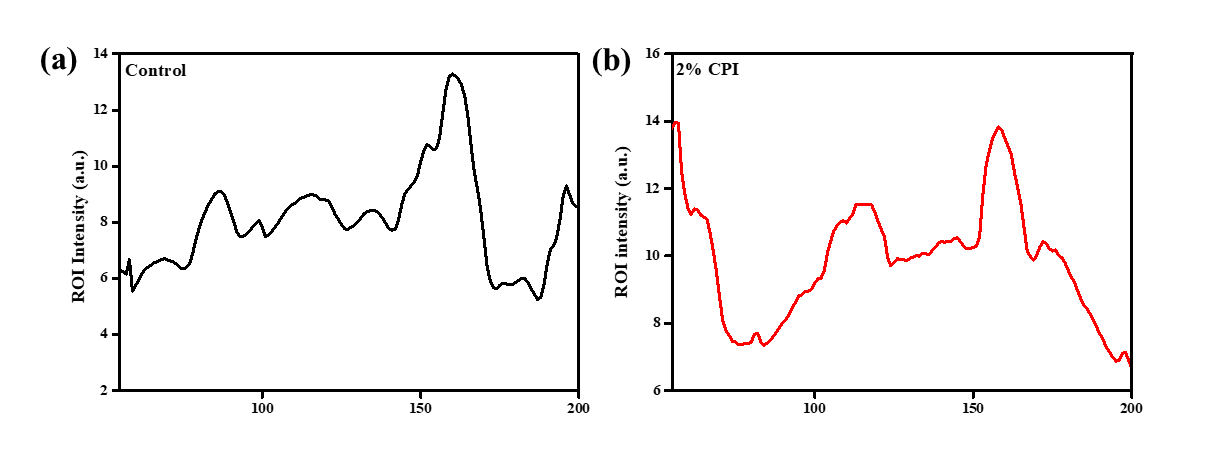
**

**S4 Fig.** ROI intensity profile of (a) control and (b) 2% CPI.

Supplement: S4 Fig — ROI intensity profile of (a) control and (b) 2% CPI. (DOCX) [file pone.0249291.s004.docx]

**Raw images:**


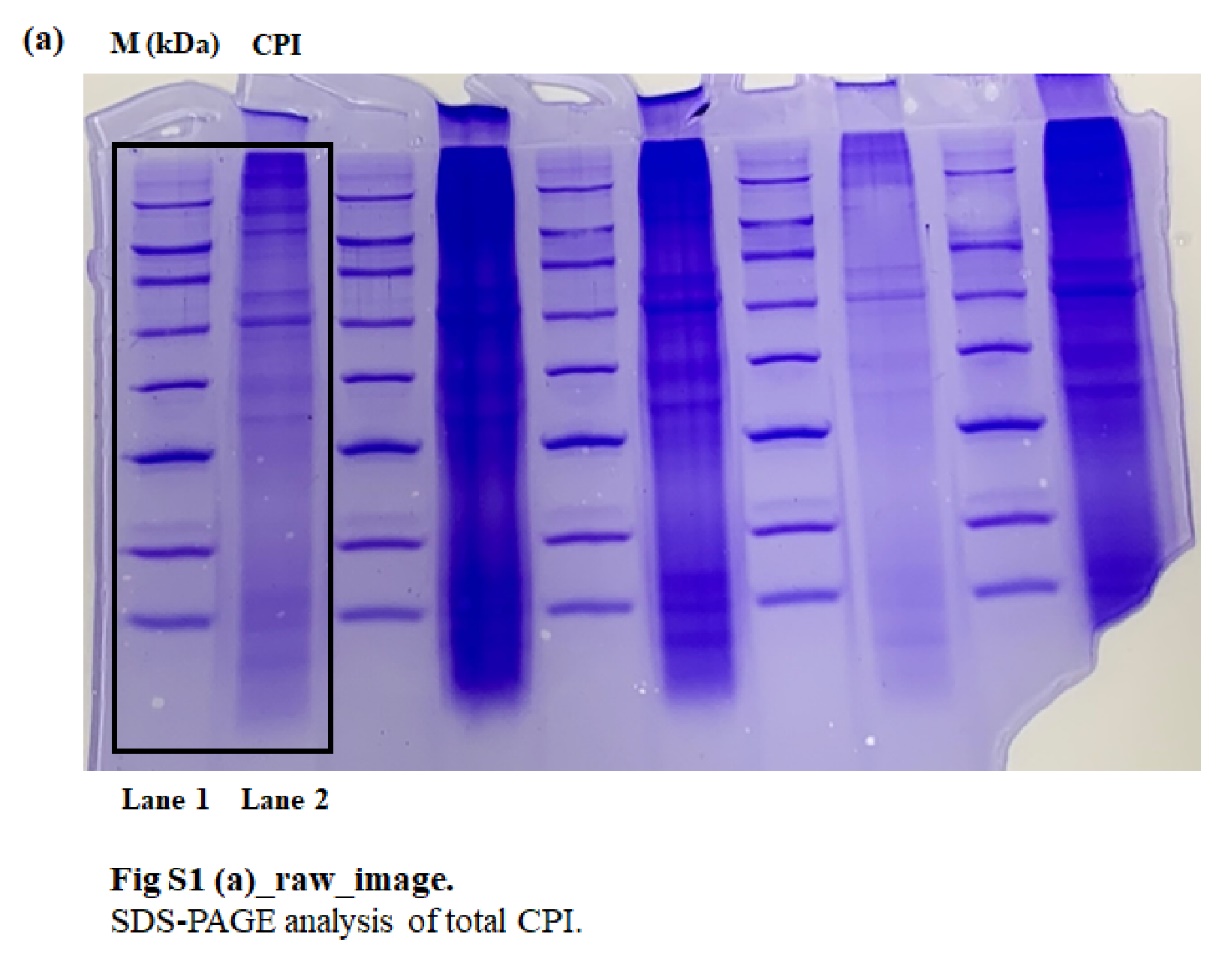


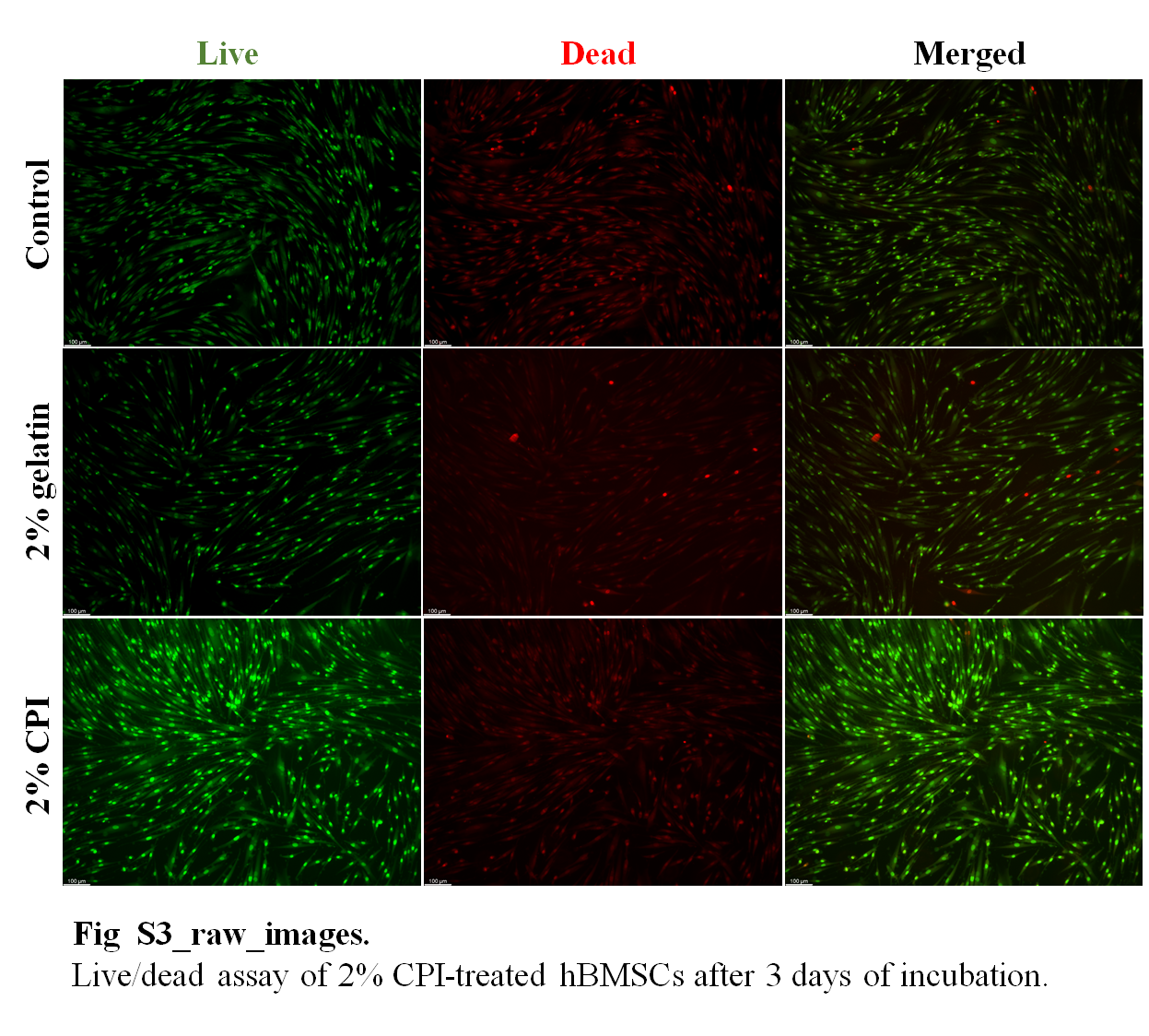


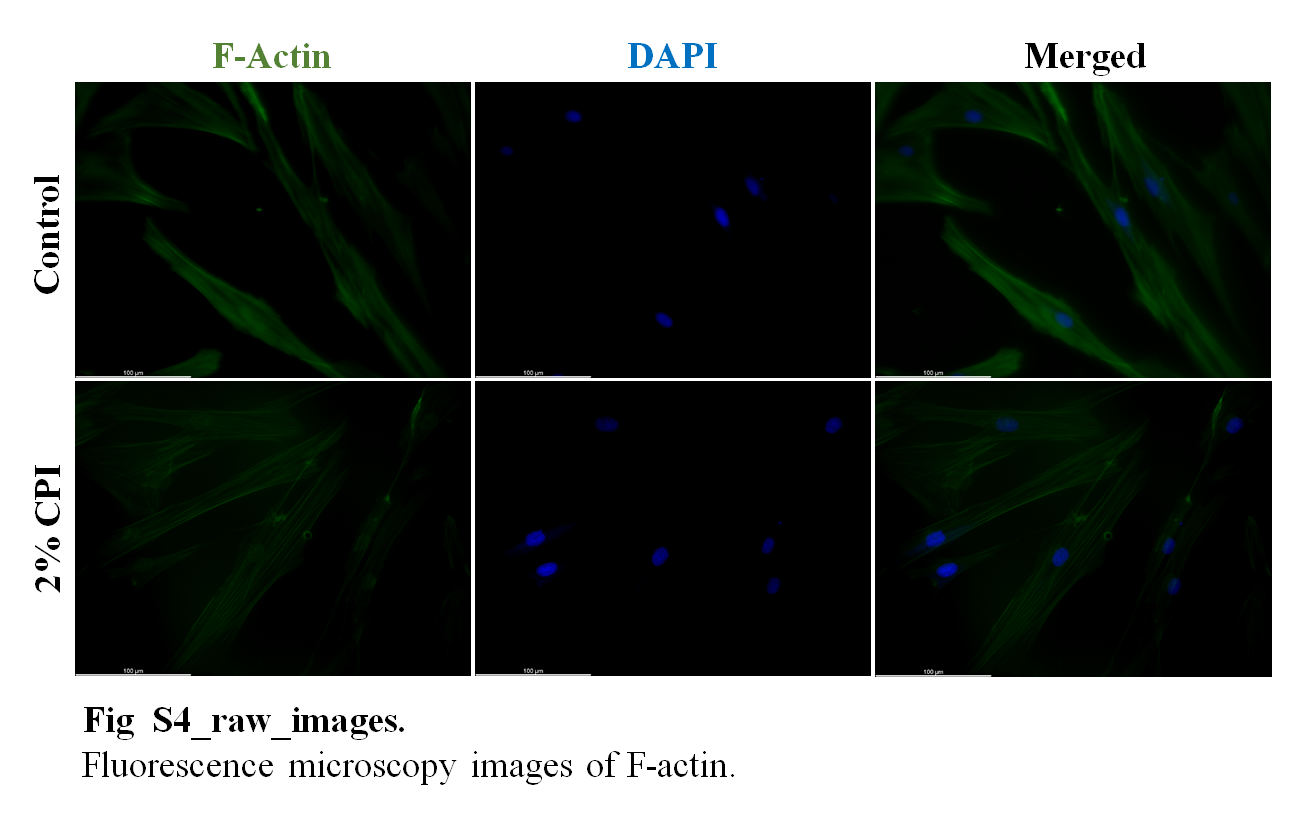


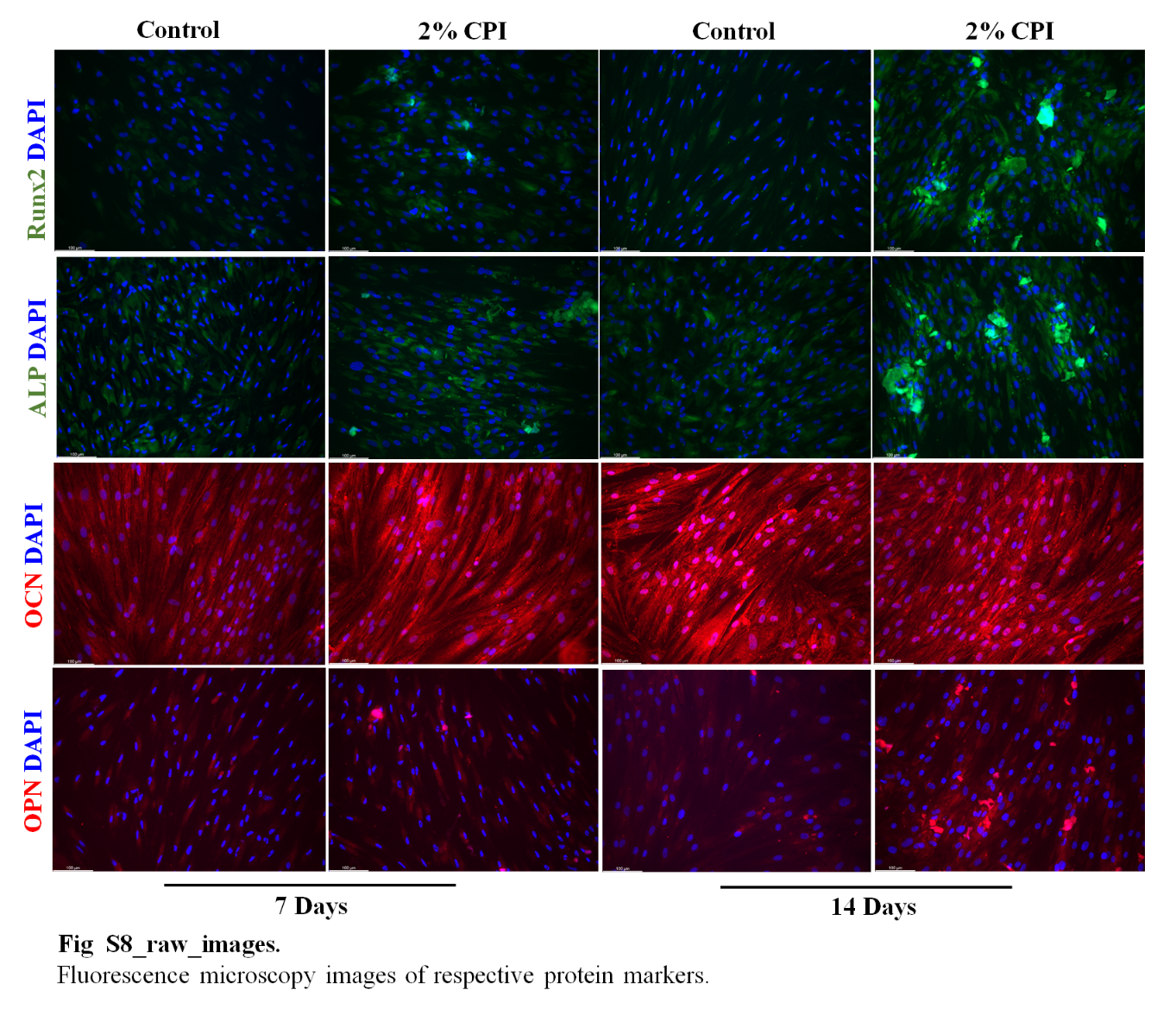

Supplement: S1 Raw images — (DOCX) [file pone.0249291.s006.docx]
