# Supplementary material for: Naturally-derived protein extract from Gryllus bimaculatus improves antioxidant properties and promotes osteogenic differentiation of hBMSCs
Source: PLoS One. 2021 Jun 2;16(6):e0249291. doi: 10.1371/journal.pone.0249291 (PMC8172014; doi:10.1371/journal.pone.0249291)
Supplement: S1 Table — (DOCX) [file pone.0249291.s005.docx]

**S1 Table.** List of antibodies used for immunofluorescence staining.

| **Antibodies** | **Dilution** | **Dye** | **Company** |
| --- | --- | --- | --- |
| Runx2 | 1:250 | AF-488 | Santa Cruz Biotechnology, USA |
| ALP | 1:250 | AF-488 | Santa Cruz Biotechnology, USA |
| OCN | 1:250 | AF-594 | Santa Cruz Biotechnology, USA |
| OPN | 1:250 | AF-594 | Santa Cruz Biotechnology, USA |
