# Supplementary material for: Naturally-derived protein extract from Gryllus bimaculatus improves antioxidant properties and promotes osteogenic differentiation of hBMSCs
Source: PLoS One. 2021 Jun 2;16(6):e0249291. doi: 10.1371/journal.pone.0249291 (PMC8172014; doi:10.1371/journal.pone.0249291)
Supplement: Attachment — Submitted filename: Response to Reviewers.docx [file pone.0249291.s007.docx]

**Response to Reviewers:**

**Reviewer 1:**

In this paper Authors aimed to evaluate the osteoinductive efficiency and antioxidative property of cricket protein isolate (CPI) on hBMSCs.

**Reply to the reviewer:** Thank you very much for reviewing our manuscript. We have made the necessary corrections based on your valuable suggestion.

**Comment #1.** English should be revised by a native speaker.

**Author's Response:** Thank you very much for your kind suggestion. We have revised the English language through the paid version of the Grammarly Premium software in the manuscript.

**Comment #2**. Introduction section should be rewritten, in particular, in the final part the authors must not describe the methods used, but emphasize the purpose and clinical applications of their research.

**Author's Response:** Thank you very much for your recommendation. We have rewritten the introduction section of the manuscript.

**Comment#3**. Images in figure 4 are not clear. It is impossible to appreciate the IF in panel b; furthermore, in panel c there is accumulation of staining in samples with 1% and 2% of CPI not evident in the positive control. To what do the authors attribute this?

**Author's Response:** Thank you very much for reviewing the manuscript. We have included the better quality of the images in **Figure 4** in the revised manuscript, which showed the cytocompatibility of the CPI in the presence of hBMSCs.

Furthermore, the morphologies of the cultured cells are presented in **Figure 5** with different staining techniques in the improved quality of the images. The accumulated stain in the treated samples are the deposited CPI and hence are absent in the positive control.

**Comment#4**. From the magnification of the images in fig 5a at 14 days the mineralization seems less than at 7 days in all the treated samples while the authors assume the opposite.

**Author's Response:** We appreciate the reviewer's valuable comment. The excessively stained area in the 7 days treated cells represent the deposited CPI. We have marked the presence of stained CPI deposited over the hBMSCs with an arrow in 7 days treated images. At 14 days' interval, most of the CPI material dissolves in the media compared to the 7 days incubated samples and is easily removed during the staining procedure.

**Comment#5**. ALP staining does not seem so pronounced.

**Author's Response:** We agree with the reviewer's observation. We have therefore removed this data from our manuscript.

**Comment#6**. Authors should also evaluate the expression of proteins involved in osteogenesis.

**Author's Response:** Thank you very much for your suggestion. We have performed an immunostaining experiment for Runx2, ALP, OCN, and OPN.

**Reviewer 2:**

Keya Ganguly and colleagues have submitted a manuscript aiming at investigating the effects of cricket derived protein isolate (CPI) on hBMSCs. They found an antioxidant effect and an improved osteoinductive efficiency.
The work has several flaws as follows:

**Reply to the reviewer:** Thank you very much for reviewing our manuscript. We have made the necessary corrections based on your valuable suggestion.

**Comment #1**. There is no information on cells adopted in experiments (cell line or primary cell culture/extraction methods)

**Author's Response:** Thank you very much for your comment. We have included this information in line no. 171-172.

**Comment #2**. In figure 4 in viability assay and cell/dead experiments should include a "positive control", in case mixed with CPI.

**Author's Response:** Thank you very much for your valuable suggestion. As cricket is an edible insect, it is well reported to be biocompatible. However, its osteoinductive efficiency is not studied much. Owing to this fact, we have included a positive control only in the mineralization test.

**Comment #3**. The differences between untreated/treated (fig. 4b) cells seems not significant, contrary to what is written in line 333.

**Author's Response:** Thank you very much for your observation. We have rearranged and enlarged **Figure 4** and indicated the dead cells with arrows.

**Comment #4**. In figure 5 After 7 days in CPI treated samples the staining is stronger than 14 days (the graph does not seem to coincide with the images)

**Author's Response:** We agree with the reviewer's valuable observation. The excessively stained area in the 7 days treated cells represent the deposited CPI. We have marked the presence of stained CPI deposited over the hBMSCs with an arrow in 7 days treated images. At 14 days' interval, most of the CPI material dissolves in the media compared to the 7 days incubated samples and is easily removed during the staining procedure.

**Comment #5**. The expression of osteogenic genes should be confirmed by corresponding protein expression.

**Author's Response:** Thank you very much for your suggestion. Based on your recommendation, we have included the immunofluorescence staining of Runx2, ALP, OCN, and OPN in **Figure 8**.

**Comment #6**. In figure 6 ALP staining is of low quality, it's hard to appreciate the differences.

**Author's Response:** Thank you very much for your observation. We have removed this data from our manuscript.

**Comment #6**. The manuscript would be benefit from native proof reading as typos and phonetical errors are spread throughout.

**Author's Response:** Thank you very much for your suggestion. We have checked the language thoroughly using the paid version of Grammarly Premium software.

(**Note**: All the efforts made during revision were written in red color in the manuscript)
