# Supplementary material for: Naturally-derived protein extract from Gryllus bimaculatus improves antioxidant properties and promotes osteogenic differentiation of hBMSCs
Source: PLoS One. 2021 Jun 2;16(6):e0249291. doi: 10.1371/journal.pone.0249291 (PMC8172014; doi:10.1371/journal.pone.0249291)
Supplement: Attachment — Submitted filename: Response to Reviewers.docx [file pone.0249291.s008.docx]

**Response to Reviewers:**

**Reviewer 1:**

The authors aimed to investigate the effects of CPI on the osteogenic differentiation of hBMSCs. The manuscript is interesting, but there are some points that must be clarified.

**Author’s response:** Thank you very much for reviewing our manuscript. We have made the necessary corrections based on your valuable suggestion.

**Comment #1.** I suggest to improve the title. First of all, it is badly written in English. I suggest to use the word biopolymer instead of biomaterial. Biopolymers are polymers produced by living organisms. Cellulose, proteins, peptides, are all examples of biopolymers used in tissue engineering. For example, I suggest a possible title as following: "Naturally derived proteins extract from Gryllus bimaculatus improves antioxidant properties and promotes osteogenic differentiation of hBMSCs".

**Author’s response:** Thank you for your kind recommendation. We agree with your suggested title and have updated the same in our revised manuscript.

[Naturally-derived protein extract from Gryllus bimaculatus improves antioxidant properties and promotes osteogenic differentiation of hBMSCs].

**Comment #2**. In figure 5a, the authors must report what the arrows indicate in the pictures.

**Author’s response:** Thank you for your comment. We have indicated the necessary information in the revised manuscript.

[**Fig. 5.** *In vitro* cytotoxicity evaluation of CPI on hBMSCs after 3 days of incubation. **(a)** optical microscopy images after Giemsa staining **(b)** fluorescence microscopy images of F-actin. The white arrows indicate the presence of stained CPI on the surface of hBMSCs].

**Comment #3**. The osteogenic differentiation must be evaluated also at 21 days.

**Author’s response:** Thank you very much for your suggestion. We have included the hBMSC differentiation using the ARS staining procedure **(Fig. 5.)** and immunohistochemistry of ALP expression **(Fig. 6.)** for 7, 14, and 21 days in our revised manuscript.

**Comment #4**. The immunofluorescence showed in figure 8 (in particular for 2%CPI) is very bad. Dye deposits are detectable and the staining is aspecific. The Authors indicate these deposits as presence of CPI on surface of hBMSCs. How can the authors confirm this? If this is, it means that the CPI is not soluble. Incomplete solubility makes CPI difficult to use. Please, clarify better this point.

**Author’s response:** Thank you very much for deeply reviewing our manuscript. We agree with your opinion. The CPI used in this study is partially soluble in DMEM at the physiological pH and hence some of the aggregated structure are visible on the surface of the cultured hBMSCs. To confirm the aggregates as CPI, we have taken the bright field images of the cells after desired treatment and is indicated in **Supplementary Fig. 3** of the revised manuscript. Thus, the fluorescence microscopy images taken at different time period also indicates the presence of CPI aggregates on the surface of cells as shown in **Fig. 8**. We completely agree that soluble protein is more convenient for cellular interaction or metabolism. However, the purpose of our study was to check the potential of crude CPI on hBMSCs osteogenic potential at physiological pH. The size and the colloidal stability of the up to 2% CPI at physiological pH was found to favor hBMSCs growth and differentiation. Based on our study, we conclude that the use of up to 2% crude CPI is favorable for hBMSCs growth.

**Comment #5**. ALP must be performed using histological staining. Moreover, the Authors stated that: “no significant difference was noted in the mean intensities among the control, and the CPI treated cells”.

**Author’s response:** Thank you very much for your suggestion. We have included the immunohistochemistry staining of ALP after 7, 14, and 21 days of CPI treatment. As the protein expression for ALP was performed using the immunocytochemistry analysis, we could confirm the successful expression of the protein in both the treated and the control cells, indicating the CPI treatment favored the expression of the osteogenic marker proteins similar to control cells.

**Comment #6**. In figure 8, the statistic is indicated using asterisks. This point must be clarified and reported in Results.

**Author’s response:** Thank you for your kind information. We have discussed this point in the “Results” section in our revised manuscript.

**Comment #7**. The Discussion must be revised, it is too long and dispersive.

**Author’s response:** Thank you for your suggestion. We have updated the discussion in our revised manuscript as per your recommendation.

**Reviewer 2:**

Risultati della traduzione
Although the study was previously not sufficiently suitable for publication on Plos One, the experiments carried out by the authors following the suggestions of the reviewers, have significantly improved the paper. Despite this, some concerns need to be addressed.

**Comment #1**. First of all, the title should be rewritten: it should be improved grammatically, with a verb that would make the meaning of the paper clearer.

**Author’s response:** Thank you very much for your valuable comment. We have changed the title in the revised manuscript as follows:

[Naturally-derived protein extract from Gryllus bimaculatus improves antioxidant properties and promotes osteogenic differentiation of hBMSCs].

**Comment #2**. Osteogenesis should also be evaluated at 21 days.

**Author’s response:** Thank you very much for your comment. We have evaluated the osteogenesis through ARS staining procedure for hBMSCs mineralization quantification **(Fig. 5.)** and ALP expression through immunohistochemistry **(Fig. 6.)** for 7, 14, and 21 days in the revised manuscript.

**Comment #3**. The discussion needs to be shortened.

**Author’s response:** Thank you very much for your kind recommendation. Based on your suggestion, we have shortened the discussion section in the revised manuscript.
